# Molecular Epidemiology of Rotavirus A Strains Pre- and Post-Vaccine (Rotarix^®^) Introduction in Mozambique, 2012–2019: Emergence of Genotypes G3P[4] and G3P[8]

**DOI:** 10.3390/pathogens9090671

**Published:** 2020-08-19

**Authors:** Eva D. João, Benilde Munlela, Assucênio Chissaque, Jorfélia Chilaúle, Jerónimo Langa, Orvalho Augusto, Simone S. Boene, Elda Anapakala, Júlia Sambo, Esperança Guimarães, Diocreciano Bero, Marta Cassocera, Idalécia Cossa-Moiane, Jason M. Mwenda, Isabel Maurício, Hester G. O’Neill, Nilsa de Deus

**Affiliations:** 1 Instituto Nacional de Saúde (INS), Maputo 1008, Mozambique; benildeantnio@gmail.com (B.M.); assucenyoo@gmail.com (A.C.); Jorfeliachilaule@gmail.com (J.C.); langajeronimo@gmail.com (J.L.); simonboene@gmail.com (S.S.B.); elda.muianga07@gmail.com (E.A.); juliassiat@yahoo.com.br (J.S.); espeguima@hotmail.com (E.G.); dmbero@gmail.com (D.B.); marti.life@hotmail.com (M.C.); idaleciacossa@yahoo.com.br (I.C.-M.); ndeus1@yahoo.com (N.d.D.); 2 Instituto de Higiene e Medicina Tropical, Universidade Nova de Lisboa, 1349-008 Lisbon, Portugal; isabel.mauricio@ihmt.unl.pt; 3 Centro de Biotecnologia, Universidade Eduardo Mondlane, Maputo 3453, Mozambique; 4 Faculdade de Medicina, Universidade Eduardo Mondlane, Maputo, P.O. Box 257, Mozambique; orvaquim@gmail.com; 5 Harris Hydraulics Laboratory, Department of Global Health, University of Washington, Seattle, WA 98195-7965, USA; 6 African Rotavirus Surveillance Network, Immunization, Vaccines and Development Program, WHO Regional Office for Africa, Brazzaville, P.O. Box 2465, Congo; mwendaj@who.int; 7 Global Health and Tropical Medicine, Instituto de Higiene e Medicina Tropical, Universidade Nova de Lisboa, 1349-008 Lisbon, Portugal; 8 Department of Microbial, Biochemical and Food Biotechnology, University of the Free State, Bloemfontein 9301, South Africa; oneillhg@ufs.ac.za; 9 Departamento de Ciências Biológicas, Universidade Eduardo Mondlane, Maputo 3453, Mozambique

**Keywords:** rotavirus type A, Mozambique vaccine surveillance, G3 genotype, Rotarix

## Abstract

Group A rotavirus (RVA) remains the most important etiological agent associated with severe acute diarrhea in children. *Rotarix*^®^ monovalent vaccine was introduced into Mozambique’s Expanded Program on Immunization in September 2015. In the present study, we report the diversity and prevalence of rotavirus genotypes, pre- (2012–2015) and post-vaccine (2016–2019) introduction in Mozambique, among diarrheic children less than five years of age. Genotyping data were analyzed for five sentinel sites for the periods indicated. The primary sentinel site, Mavalane General Hospital (HGM), was analyzed for the period 2012–2019, and for all five sites (country-wide analyses), 2015–2019. During the pre-vaccine period, G9P[8] was the most predominant genotype for both HGM (28.5%) and the country-wide analysis (46.0%). However, in the post-vaccine period, G9P[8] was significantly reduced. Instead, G3P[8] was the most common genotype at HGM, while G1P[8] predominated country-wide. Genotypes G9P[4] and G9P[6] were detected for the first time, and the emergence of G3P[8] and G3P[4] genotypes were observed during the post-vaccine period. The distribution and prevalence of rotavirus genotypes were distinct in pre- and post-vaccination periods, while uncommon genotypes were also detected in the post-vaccine period. These observations support the need for continued country-wide surveillance to monitor changes in strain diversity, due to possible vaccine pressure, and consequently, the effect on vaccine effectiveness.

## 1. Introduction

Group A rotavirus (RVA) remains the most important etiological agent associated with severe acute diarrhea in children worldwide [1,2,3]. In 2016, RVA was estimated to cause more than 128,000 deaths among children younger than five years throughout the world, with more than 104,000 deaths occurring in sub-Saharan Africa [3].

RVA is a non-enveloped, double-stranded RNA virus. The segmented genome has 11 gene segments which encode six structural viral proteins (VP1, VP2, VP3, VP4, VP6, and VP7) and six non-structural viral proteins (NSP1, NSP2, NSP3, NSP4, and NSP5/6) [4,5,6]. The viral capsid is composed of three concentric layers which encapsulate the 11-segmented genome. The outer layer is composed of the viral spike protein, protease-sensitive VP4, and glycoprotein VP7. A dual typing system for RVA is based on the gene segments encoding VP4 (P genotypes) and VP7 (G types). The rotavirus classification-working group has identified 36 G and 51 P genotypes globally in humans and in the young of many mammalian and avian species [7,8,9,10]. Six G types (G1, G2, G3, G4, G9, G12) and 3 P types (P[8], P[4], P[6]) predominate globally [11,12,13,14], although in Africa and Asia genotypes, such as G5, G6, and G8, are also described as important [15]. The six most frequently reported G/P combinations associated with infections in humans worldwide are G1P[8], G2P[4], G3P[8], G4P[8], G9P[8], and G12P[8] [10,11,12,13,14,16].

In 2009, the World Health Organization (WHO) recommended the introduction of rotavirus vaccines in national immunization programs worldwide and particularly in countries with a high under-five mortality rate associated with diarrhea [17]. The WHO has coordinated the Global Network of Rotavirus surveillance (GNRS) since 2006 to support countries with evidence-based decision-making [10]. Mozambique has actively participated in WHO rotavirus surveillance since 2016. Continuous surveillance of circulating genotypes, as well as the monitoring of disease burden, is important to evaluate the effectiveness of rotavirus vaccines.

Before the introduction of rotavirus vaccines, a high rotavirus disease burden was reported in particular the southern Mozambican region. However, due to a lack of surveillance, no information was available from the center and northern regions of the country [18,19,20]. In the Global Enteric Multicenter Study (GEMS), which determined the burden and etiology of diarrhea in children under five years of age in four sub-Saharan African and three Asian countries, Mozambique had the highest attributable fraction (27.0%) of rotavirus-associated diarrhea among infants [20]. In Mozambique, the prevalence of rotavirus in under-five year old children from urban (Maputo City) and rural (Manhiça District) areas in 2012 and 2013 was higher than 40.0% [19]. A lower infection rate (24.0%) was, however, reported in 2011 in Gaza province, a rural area [18]. Data from the National Surveillance of Diarrhea also showed a high rotavirus infection rate of 40.2% and 38.3% in 2014 and 2015, respectively, before vaccine introduction in Mozambique [21]. The monovalent vaccine, *Rotarix*^®^ (GlaxoSmithKline, Rixensart, Belgium), was introduced into the Expanded Program on Immunization of Mozambique in September 2015. Since then, the prevalence of rotavirus infections of 12.2% and 13.5% in 2016 and 2017, respectively, has been reported [21].

The evolution of RVA through the accumulation of point mutations, gene reassortment, recombination and interspecies transmission [5,22,23], call for rotavirus strain surveillance to elucidate the effect, if any, of rotavirus vaccine usage on the circulation of rotavirus genotypes in Mozambique. The main objective of the present study was to evaluate the distribution of rotavirus genotypes prior to (2012–2015) and following (2016–2019) rotavirus vaccine introduction in Mozambique, among diarrheic children less than five years of age.

## 2. Results

### 2.1. Comparison of Rotavirus G- and P-Types in Mozambique Pre- and Post-Vaccine Introduction

From May 2014 to December 2019, a total of 1736 diarrheal stool samples were collected in five sentinel sites as part of the National Surveillance of Diarrhea program in Mozambique. Of these stool samples, 468 tested positive for RVA by ELISA (27.0%) (Supplementary Table S1). A total of 94.0% (440/468) of these samples were genotyped, *n* = 245 from Maputo (HGM and HJM), *n* = 149 from Nampula (HCN), *n* = 34 from Quelimane (HGQ) and *n* = 12 from Beira (HCB) (Supplementary Table S2). During the pre-vaccine period (2014–2015) a total of 246 samples were genotyped and in the post-vaccine period (2016–2019) 194 samples (Supplementary Table S1). In total, 6.0% (28/468) were excluded from genotyping as an insufficient amount of sample was available.

For HGM, a total of 200 genotyped samples corresponded to the pre-vaccine period (2012–2015) and 43 to the post-vaccine period (2016–2019) (Supplementary Table S3). The samples from the pre-vaccine period also included 91 genotyped samples collected at HGM between 2012 and 2013 from a cross-sectional study [24] to extend the analyses for this particular site (Supplementary Table S3).

The analyses for HGM showed that G9 was the most prevalent G type (30.5%) in the pre-vaccine period (*n* = 200), but was significantly reduced to 9.3% during the post-vaccination period (*n* = 43). Similarly, G12 was also significantly reduced (from 18.5% to 2.3%) (Table 1). In contrast, during the pre-vaccination period, no G3 strains were detected; but during the post-vaccine period, the genotype was the most prevalent genotype (48.8%). Interestingly, a small increase in prevalence was observed for the G1 genotype, although this increase was not statistically significant (Table 1).

P[8] was the most predominant P type in the pre-vaccine period (54.0%) (Table 1), as well as the post-vaccine period (51.2%). Only P[4] (37.2%) (Table 1) had a statistically significant increase during the post-vaccine period (*p* < 0.001). No mixed P types were detected during the post-vaccine period.

When all five sentinel sites (including HGM) were analyzed for the period of 2015–2019, a similar trend was observed for the G9 genotype. During the pre-vaccine period (*n* = 213), G9 was the most prevalent G type at 49.3%, but a significant reduction for G9 (25.3%) was reported during the post-vaccine period (*n* = 194). The emergence of G3 was also observed, becoming the most prevalent genotype, although only at 26.3% (Table 2). In contrast, a reduction in the prevalence of the G1 genotype was observed (31.5% reduced to 21.6%) for all five sentinel sites.

During the pre-vaccine period, P[8] was the most frequently detected P genotype accounting for 85.4% of all genotypes detected (Table 2). However, this high frequency was significantly reduced in the post-vaccination period to less than half (39.2%). An increase in the detection of P[6] (19.1%) and P[4] (36.6%), from almost undetectable, were recorded during this period (Table 2).

Analyses of the data recorded for samples collected at the HGM, showed a slight increase in the odds ratio for G1 type from pre-vaccine to the post-vaccine period of 1.47 times (OR = 1.47 95CI = 0.59–3.44, *p* > 0.330), but a decrease in the odds ratio for genotypes G12 of 90.0% (OR = 0.10, 95CI = 0.003–0.66, *p* < 0.008) and G9 of 77.0% (OR = 0.23, 95CI = 0.01–0.69, *p* < 0.004), respectively (Table 1). Considering all the sentinel sites, a significant decrease was observed in the odds ratio for G1 genotype from pre-vaccine to the post-vaccine period of 40.0% (OR = 0.60, 95CI = 0.37–0.96, *p* < 0.030), as well as a reduction for G9 of 65.0% (OR = 0.35, 95CI = 0.22–0.54, *p* < 0.001) (Table 2).

A reduction for genotype P[8] from pre-vaccine to the post-vaccine period was also observed at HGM (11.0%, OR= 0.89, 95CI= 0.43–1.83, *p* > 0.740, Table 1), as well as for the country-wide sentinel sites (90.0% (OR = 0.10, 95CI = 0.06 to 0.16, *p* < 0.001, Table 2). In contrast, a significant increase in the odds ratio of genotype P[4] of 3.23 times (OR = 3.23, 95CI = 1.44–7.04, *p* < 0.001) was observed at the HGM (Table 1). Analyses for all the sentinel sites showed a high prevalence for P[4] during the post-vaccine period (36.6%) compared to the pre-vaccine period (0.5%).

### 2.2. Comparison of G/P Genotype Combinations in Mozambique Pre- and Post-Vaccine Introduction

At HGM, the most predominant combinations during the pre-vaccine period were G9P[8] (28.5%), G1P[8] (17.0%), G12P[6] (13.0%) and G2P[4] (10.0%), comprising a total of 68.5% of all genotypes analyzed (Table 3). During the post-vaccine period, G1P[8] (20.9%) was still one of the predominant combinations, although G3P[8] and G3P[4] strains were detected at 25.6% and 18.6%, respectively (Table 3). A significant reduction in G9P[8] detection was observed following vaccine introduction (*p* < 0.001). Instead, the G9 genotype was now detected in combination with P[4] and P[6] both at a frequency of 4.7% (Table 3).

The most frequent G/P combinations observed for all the sites participating in the National Surveillance of Diarrhea program during the pre-vaccine period were G9P[8] and G1P[8] at 46.0% and 31.0%, respectively. These combinations comprised a total of 77.0% of all genotypes analyzed (Table 4).

In the post-vaccine period, G1P[8] remained the most frequent G/P combination, but at a reduced frequency of 20.6%. G2P[4] (at a slightly higher frequency) and G2P[6] (similar frequency as in 2015) were, again, detected in the post-vaccine period. Similar to the analysis for HGM, G3 in combination with P[4] (14.4%) and P[8] (9.8%) were detected during the post-vaccine period, together with G9P[4] (12.4%) and G9P[6] (8.8%). Mixed infections, as determined with RT-PCR, was detected for 6.2% of the samples (Table 4).

Analyses for HGM showed an increase in the odds for G1P[8] at 1.29 times (95CI = 0.50–3.07, *p* > 0.54), but a significant decrease in the odds ratio for G9P[8] at 94.0% (OR = 0.06, 95CI = 0.002–0.40, *p* < 0.001) (Table 3).

In contrast, a significant decrease in the odds ratio for all the sentinel sites was observed for G1P[8] at 42.0% (OR = 0.58, 95CI= 0.36–0.93, *p* < 0.020) and G9P[8] at 96.0% (OR = 0.04, 95CI= 0.02–0.10, *p* < 0.001) (Table 4).

### 2.3. Yearly Distribution of Rotavirus Genotypes at the Mavalane General Hospital (HGM) and National Surveillance Sites

As reported before, G12P[6] (28.6%) and G2P[4] (23.1%) were the most predominant genotype combinations at HGM during 2012–2013 [24]. In 2014 and 2015, G1P[8] and G9P[8] with 84.8% and 73.7%, respectively, were detected at the highest frequencies. In 2016, during the post-vaccine period, the most frequent genotype was G1P[8] with 66.7%. The emergence of new genotypes was observed in 2016 (G3P[4]), which increased in 2017, to the most prevalent genotype (25.0%) followed by G1P[8] (18.8%) (Table 5). In 2018, G3P[8] and G3P[4] became the most prevalent genotype combinations with 36.4% and 27.3%, respectively. Finally, in 2019, only G3P[8] were detected at the HGM. No G1P[8] strains were, therefore, detected in 2018 and 2019 (Table 5).

Since data is available for only one year for all five participating sentinel sites during the pre-vaccine period, yearly analysis for the national surveillance sites are presented from 2015–2019. The results showed that in 2015 the most frequent G/P combination was G9P[8] (46.0%), followed by G1P[8] (31.0%). In 2016, G1P[8] was detected at the highest frequency (43.6%) (Table 6). Other genotype combinations, such as G2P[6] (17.9%), G9P[6] (12.8%), G9P[4] (7.7%), and G3P[4] (2.6%), were also observed in 2016 (Table 6). These results were comparable to those from HGM.

In 2017, G1P[8], as well as G9P[4], were detected at similar frequencies (19.2%), while G3P[4] was detected at 13.5% (Table 6). In 2018 and 2019, G3P[4] and G3P[8] became the most frequently detected genotype combination with 38.7% and 60.0%, respectively (Table 6). G3 was also observed in combination with P[4] (13.5%) and P[6] (1.9%) in 2017, whereas G1P[8] genotype was not detected in 2018, although this genotype was detected at 15.0% in 2019 (Table 6). The results reported for all the sentinel sites participating in the National Surveillance of Diarrhea program is comparable to that observed for HGM for the reporting period (2015–2019), except that G1P[8] was not detected in 2019 for HGM.

### 2.4. Geographical Distribution of Rotavirus Genotypes

A variation in rotavirus genotypes between the five sentinel sites in Mozambique was observed (Supplementary Table S4).

In the pre-vaccine period (2015), it was observed that G1P[8] occurred in all regions included in this study, with the highest frequency (78.0%) detected in the northern region, at Nampula (HCN) (Supplementary Table S4). In contrast, the G9P[8] genotype combination was mostly detected in the southern region, Maputo (HGM and HJM) at 68.8%. Other uncommon genotypes, such as G2P[6], were mostly detected at Nampula at 10.2% but were not detected in Quelimane (HGQ) or Beira (HCB) (Supplementary Table S4). Similarly, in the post-vaccine period (2016–2019), the combination G1P[8] was observed across the country. In 2016 at Maputo and Nampula, G1P[8] was the most prevalent genotype with 66.7% and 43.5%, respectively. The G1P[8] genotype was, however, also detected in Quelimane and Beira, which had small sample sizes.

In 2017 the genotype combination G3P[4] was the most prevalent (29.7%) in Maputo, while in Nampula and Quelimane G9P[4] and G9P[6] were the most prevalent at 32.7% and 35.7%, respectively. In 2018 and 2019, the G3 genotypes were predominantly detected in Maputo and Quelimane in combination with P[4] and P[8]. In Nampula and Beira, G3 was detected in combination with P[4] (Supplementary Table S4).

## 3. Discussion

Before rotavirus vaccine introduction in Mozambique, RVA surveillance studies focused in the southern region of the country [18,19,20]. Instituto Nacional de Saúde (INS) initiated national RVA surveillance in the southern region of Mozambique in 2014, which was expanded to other regions (center and north) in 2015. Following the country-wide introduction of *Rotarix*^®^ in September 2015, its impact has been monitored and a substantial reduction in the prevalence of RVA infection rate to 12.2% and 13.5% in 2016 and 2017, respectively, was reported [21]. Since the country is vast, it is important to expand strain surveillance to include the entire country.

In the present analysis, rotavirus surveillance that form part of the National Surveillance of Diarrhea during 2014–2019, as well as data from a cross-section study at the HGM from 2012 and 2013, are reported [24].

During the surveillance at HGM (2012–2019), as well as country-wide sentinel sites (2015–2019), variations in the prevalence of genotypes in the pre- and post-vaccine periods were observed. Genotypes G9 and P[8] were consistently the most prevalent in the pre-vaccine period and in the post-vaccine period, genotypes G3 and P[8] were the most prevalent. However, the proportion of P[8] was reduced, and the prevalence of genotype P[4] increased. These results suggest that genotype prevalence can vary from year to year pre- or post-vaccination in Mozambique.

When comparing the most predominant G/P combinations before and after vaccine introduction at the HGM, G9P[8] was the most predominant genotype combination in the pre-vaccine period, while G1P[8] was the most prevalent genotype combination in the post-vaccine period. The country-wide surveillance also revealed a decreased odds ratio for G9P[8] after the introduction of the vaccine. However, this reduction was accompanied by the emergence of G9P[4] and G9P[6], especially in the northern part of Mozambique, after vaccine introduction. Finally, the emergence of G3P[4] and G3P[8] was also observed. These results showed that in this early phase of rotavirus strain surveillance, it is not clear whether these variations in genotype combinations between both periods were due to the rotavirus vaccine or simply natural variation in genotype frequency. Our results are consistent with previously published studies, as a number of countries from Africa, Europe and America reported a variation in the strain diversity between the two periods [16,25,26,27,28,29,30].

Countries that introduced the monovalent *Rotarix*^®^ vaccine similar to Mozambique, reported a decline of genotype G1P[8] with a concurrent rise in other combinations in the post-vaccine period. For example, South Africa reported an increase in non-G1P[8] strains [25]. In contrast, in Malawi, the reduction of G1P[8] was not significant [27]. In Ghana, G1P[8] returned as one of the dominant strains in the fourth year post-vaccine introduction [26]. Other studies reported from England, Brazil, Belgium, Scotland, a decline in the proportion of G1P[8] with a rise in the proportion of heterotypic strains, such as G2P[4], was observed [28,29,30,31].

Additionally, Belgium reported a slightly lower vaccine effectiveness against G2P[4], and in Malawi, a lower vaccine effectiveness against G2 strains than G1 strains was reported [27].

In our analyses, HGM, with at least four years pre-vaccine data showed a slight increase of G1P[8] after vaccine introduction, although in the country-wide analyses the G1P[8] prevalence was reduced. This needs careful interpretation, due to the difference in the number of years in the pre-vaccine period, one of the limitations of this analysis.

Regarding the variation in the prevalence of some uncommon genotypes (e.g., G9P[4] G9P[6], G3P[4], G3P[6]) detected after vaccine introduction in Mozambique, it is important to mention that a number of studies in Africa [16,25,32] and Asia (India and Japan) also reported these uncommon genotypes before vaccine introduction in low frequency [33,34]. These uncommon genotypes, apart from G9P[6], were also observed in Ireland before vaccine introduction [35,36,37]. However, a study conducted in Ghana reported the emergence of G9P[4] at a low frequency only during the fourth rotavirus season after vaccine introduction [26].

The emergence of the genotype combinations G3P[4], detected in 2016, 2017, 2018, and G3P[8] in 2018 and 2019 was observed in Mozambique. These strains were also reported in the same period in Botswana after vaccine introduction in 2012 [38]. Botswana also reported an outbreak of G3P[8] in 2018 [39]. In addition, several countries reported G3 in combination with P[4] and P[8] during the 12th African Rotavirus Symposium 2019 [40,41,42]: Malawi (introduced vaccine in 2012, reported G3P[8] in 2018), South Africa (introduced vaccine in 2009, reported G3P[4] in 2015–2016), Kingdom of Eswatini (introduced vaccine in 2015, reported G3P[8] in 2018). These observations suggest that G3 strains were circulating in Southern Africa during 2015–2018, with a sharp increase in 2018. Around the world, the emergence of genotype G3P[8] and equine-like G3P[8] in 2013 in Australia and re-emergence of G3P[8] were observed in Brazil in the post-vaccine introduction [43,44,45]. The European Rotavirus Network (EuroRotaNet) reported 2017–2018 for the first time since inception, G3P[8] as the most prevalent strain [28].

Temporal variation of rotavirus strains was observed in Mozambique, in particular in the model site, Mavalane General Hospital (HGM), as data from a cross-sectional study that characterized rotavirus strains at the HGM from 2012 and 2013 [24], was combined with data generated at the same site as part of the National Surveillance program with its inception in 2014. As already mentioned, G12P[6] was the most predominant genotype in 2012, and in 2013, G2P[4] was the most prevalent [24]. In a similar time period, G12P[6]was also reported in the Manhiça District, while in 2011 in the Chókwè district, G12P[8] was the most prevalent genotype [18,24]. These results suggest circulation of G12 during 2011–2012 in southern Mozambique. The G12 genotype was detected at a prevalence of almost 20% in Sub-Saharan Africa during 2012–2013 [10,16]. In 2013 the G2P[4] was the predominant genotype in the Manhiça district [24] and also in South Africa in 2013 [46]. A shift in genotypes was observed in 2014 and 2015 when mostly G1P[8] and G9P[8] strains were detected.

In the post-vaccine period (2016–2019), G9P[8] was replaced by G1P[8] in 2016, while in 2017, G3P[4] was the most predominant followed by G1P[8]. In 2018 and 2019, no G1P[8] strains were detected; instead, the G3P[8] genotype was the most prevalent. The G3P[8] genotype combination is one of the most prevalent strains associated with human rotavirus infection globally [11,12,13,14]. However, G3P[4], which is considered an uncommon combination, was also detected. Studies published previously in Mozambique during the pre-vaccine period did not detect these strains. These temporal analyses clearly showed a yearly variation of rotavirus strains, complicating the assessment of vaccine introduction impact on changes in strain diversity [11,12,13,14]. These observations are further supported by data generated by the National Surveillance of Diarrhea that also showed a temporal variation of rotavirus strains and may rather represent the natural variation in rotavirus strains.

Evaluation of strains detected at the various sentinel sites between 2015–2019, showed that G1P[8] was detected at all sentinel sites, albeit at a variation in frequency. It is interesting to note that G9P[8] occurred mostly in Maputo (HGM and HJM) in the southern region of the country, while G9 in combination with P[4] and P[6] were observed mostly in the north, Nampula (HCN), and central region, Quelimane (HGQ). The occurrence of G2P[6] was mostly observed in Nampula. The emergence of G3 strains was, however, detected at all sites under surveillance suggesting that the occurrence of these strains was not location bound. Differences in the geographical distribution of genotypes within a country was previously reported [11].

Various challenges and limitations were experienced during the study. These include logistical issues, which led to a delay in the start of surveillance at some sentinel sites. The study was limited by its small sample size; therefore, it was not possible to perform in-depth temporal analyses by the site to access the genetic variability of strains. Furthermore, bias in strain diversity is possible since a low number of strains were characterized at some sentinel sites. Extended pre-vaccine genotyping data (four years) was available for only one sentinel site, whereas only one year genotyping data were available for the remainder of the sentinel sites.

Despite the circulation of diverse rotavirus strains and the emergence of some genotypes, the National Surveillance of Diarrhea reported a reduction in rotavirus prevalence during the early impact study of the rotavirus vaccine after vaccine introduction.

The whole genome characterization of rotavirus strains circulating pre- and post-vaccine introduction will be useful to evaluate any potential vaccine-induced selection of specific antigenic profiles. Moreover, with recent reports related to the emergence of double-reassortant G1P[8] on a DS-1–like genetic backbone [47,48,49], whole-genome characterization will be important for strains surveillance.

## 4. Materials and Methods

### 4.1. Study Population and Stool Samples Collection

RVA positive samples, as tested by Enzyme-Linked Immunosorbent Assay (ELISA), were included. Samples were obtained from children under five years of age suffering from moderate-to-severe acute and non-acute diarrhea. These samples were collected as part of an ongoing hospital-based diarrhea surveillance program, called the National Surveillance of Diarrhea (ViNaDia) that commenced in May 2014. Samples were included for this study up to December 2019. In addition, data from a cross-sectional study conducted at the Mavalane General Hospital (HGM) from January 2012 to September 2013 were also included in the analyses [24].

**Figure 1:**
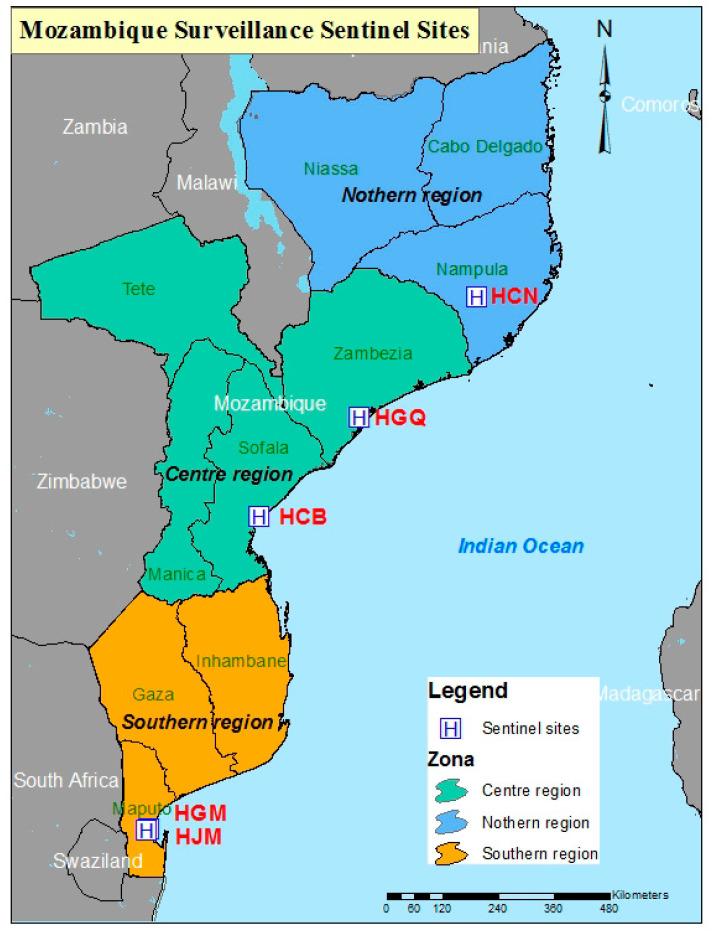
Map of Mozambique indicating the geographical location of study sites. Abbreviations for hospitals are indicated in red. HGM (Mavalane General Hospital), HJM (Jose Macamo General Hospital), HCB (Beira Central Hospital), HGQ (Quelimane General Hospital) and HCN (Nampula Central Hospital).

The National Surveillance of Diarrhea in children was led by the “Instituto Nacional de Saúde” (INS), started in May 2014 at the Mavalane General Hospital (HGM, first sentinel site) in the Maputo province (Figure 1). In March 2015, José Macamo General Hospital (HJM), also Maputo Province, and Nampula Central Hospital (HCN), in Nampula province in the northern region of the country were added. Surveillance was extended to two additional sentinel sites in June 2015: Beira Central Hospital (HCB) in Sofala Province and Quelimane General Hospital (HGQ) in the Zambézia province (Figure 1). Since 2016, Mozambique participates and actively report data to the WHO African Rotavirus Surveillance Network (ARSN). ARSN monitors rotavirus infection in children with severe acute watery diarrhea as part of a hospital-based sentinel-site surveillance program.

In the surveillance at HGM and HJM samples were collected and immediately transferred to the INS laboratory, while at HCB, HCN and HGQ, samples were collected and stored at −20 °C. Samples were transported on a weekly basis on dry ice to the INS laboratories located in Maputo City for testing and stored in −70 °C as previously described [21]. The cross-sectional study was conducted at the Centro de Investigação em Saúde de Manhiça (CISM). The sampling, testing procedures, clinical, socio-demographic information and characterization of rotavirus strains, as previously described [19,24].

### 4.2. Ethical Approval

The National Surveillance of Diarrhea in children protocol was reviewed and approved by the Mozambican National Committee on Bioethics for Health (CNBS) (reference N°: 348/CNBS/13; IRB00002657), as well as the rotavirus cross-sectional study (reference N°286/CNBS/10; IRB00002657).

### 4.3. Laboratory Testing

#### 4.3.1. Rotavirus Detection and RNA Extraction

All samples analyzed, were tested for rotavirus using the commercial Enzyme-immuno-sorbent assay (ELISA) kit (Prospect, Oxoid Ltd., Hampshire, UK) following the manufacturer’s instructions. Total RNA was extracted from ELISA-positive samples using the QIAamp Viral RNA protocol (QIAGEN, Hilden, Germany), and stored at −70 °C.

#### 4.3.2. Reverse Transcriptase (RT) and G/P Typing PCR

Extracted RNA (8 µL) was reverse transcribed using Con2/Con3 for the partial VP4-encoding gene (VP8*, 876 bp) and sBeg9/End9 for the VP7-encoding gene. G genotypes were subsequently determined using a multiplex semi-nested PCR as described before [24]. Specific primers that identified the VP7-encoding gene with the following G genotypes: G1, aBT1; G2, aCT2; G3, aET3 or mG3; G4, aDT4; G8, aAT8; G9, aFT9, or mG9; G12, G12b; G10, mG10 in combination with the common primer RVG9 were used as described previously [50,51,52].

Similarly, Con3 was used in combination with specific primers that identify P genotypes: P[8], 1T-1D or 1T-1v; P[4], 2T-1; P[6], 3T-1; P[9], 4T-1, and P[10], 5T-1, P[11], mp11, P[14], P4943, as described previously [53,54,55]. The PCR product was analyzed using 2% agarose gel electrophoresis, stained with ethidium bromide and visualized under ultraviolet illumination.

### 4.4. Data Management and Statistical Analyses

The rotavirus vaccine, *Rotarix*^®^, was introduced in September 2015 in Mozambique. Therefore, the pre-vaccine period was considered to be before December 2015, due to logistical problems associated with vaccine introduction across the country.

The genotyping data from the primary sentinel site, Mavalane General Hospital (HGM), was analyzed separately from other sites because data at this site was available from 2012 and other sites from 2015.

Frequencies of identified genotypes are reported. To assess the magnitude of change in genotypes from the pre- to post-vaccine periods, unadjusted odds ratios (OR) and their 95% confidence intervals (95CI) were computed. In this analysis, the genotype was the dependent variable and time the predictor. All statistical analysis was conducted using Stata software version 15.0 (Stata Corp., College Station, TX, USA). A *p*-value of <0.05 was considered statistically significant.

## 5. Conclusions

This is the first report describing the circulation of rotavirus genotypes in three regions of Mozambique. A comparison between the pre- and post-vaccine introduction periods showed a shift in circulating genotypes following vaccine introduction. However, due to the short surveillance period, it is not clear if the observed changes were due to the introduction of the vaccine or a consequence of natural strain variation. In addition, the emergence of unusual strains, such as G3P[4] and G3P[8], was also observed, which support the need for continued country-wide surveillance to monitor changes, due to possible vaccine pressure, and consequently, the effect on vaccine effectiveness.

## Figures and Tables

**Table 1:** Prevalence of G and P types at Mavalane General Hospital pre- and post-vaccine introduction in Mozambique (2012–2019).

^1^ G Type	Pre-Vaccine		Post-Vaccine		OR (95% CI)	*p*-Value
	^5^ 2012–2015		2016–2019			
	n	%	n	%		
G1	34	**17.0**	10	**23.3**	1.47 (0.59–3.44)	0.330
G12	37	**18.5**	1	2.3	0.10 (0.003–0.66)	0.008
G2	25	**12.5**	1	2.3	0.16 (0.004–1.08)	0.054
G3	0	0.0	21	**48.8**	-	-
G8	6	3.0	1	2.3	0.76 (0.02–6.61)	0.810
G9	61	**30.5**	4	9.3	0.23 (0.01–0.69)	0.004
^2^ Mix G	10	5.0	1	2.3	0.45 (0.01–3.30)	0.440
^3^ Gx	27	13.5	4	9.3	0.65 (0.16–2.04)	0.450
Total	200	100.0	43	100.0	-	-
^1^ P type	-	-	-	-	-	-
P[4]	31	15.5	16	**37.2**	3.23 (1.44–7.04)	<0.001
P[6]	32	16.0	3	7.0	0.39 (0.07–1.36)	0.120
P[8]	108	**54.0**	22	**51.2**	0.89 (0.43–1.83)	0.740
Mix P	8	4.0	0	0.0	-	-
^4^ P[x]	21	10.5	2	4.7	0.42 (0.05–0.82)	0.230
Total	200	100.0	43	100.0	-	-

^1^ It is not possible to calculate the Odds-ratio (OR) for cells with a value of 0; ^2^ Mix G: 2012–2015: G12G8 (2.0%), G12G9 (1.5%), G9G2 (1.5%); 2016–2019: G12G3 (2.3%); ^3^ x—refers to strains that were non-typeable for G; ^4^ x—refers to strains that were non-typeable for P; ^5^ Reference category: Pre-vaccine; Bold: The most prevalent genotypes per period.

**Table 2:** Prevalence of G and P types at five sentinel sites in Mozambique during surveillance pre- and post-vaccine introduction (2015–2019).

^1^ G Type	^5^ Pre-Vaccine		Post-Vaccine		OR (95% CI)	*p*-Value
	2015		2016–2019			
	n	%	n	%		
G1	67	**31.5**	42	**21.6**	0.60 (0.37–0.96)	0.030
G12	2	0.9	2	1.0	1.18 (0.08–15.29)	0.930
G2	10	4.7	11	5.7	1.22 (0.46–3.28)	0.660
G3	0	0	51	**26.3**	-	-
G8	0	0	3	1.5	-	-
G9	105	**49.3**	49	**25.3**	0.35 (0.22–0.54)	<0.001
^2^ Mix G	0	0	12	6.2	-	-
^3^ Gx	29	13.6	24	12.4	0.90 (0.48–1.66)	0.710
Total	213	100.0	194	100.0	-	-
^1^ P type	-	-	-	-	-	-
P[4]	1	0.5	71	**36.6**	-	-
P[6]	10	4.7	37	19.1	4.78 (2.23–11.10)	<0.001
P[8]	182	**85.4**	76	**39.2**	0.10 (0.06–0.16)	<0.001
^4^ P[x]	20	9.4	10	5.2	0.57 (0.23–1.32)	0.100
Total	213	100.0	194	100.0	-	-

^1^ It is not possible to calculate the Odds-ratio (OR) for cells with a value of 0; ^2^ Mix G—2016–2019: G12G3 (0.5%), G2G1 (0.5%), G3G1 (2.1%), G9G3 (3.1%); ^3^ x—refers to strains that were non-typeable for G; ^4^ x—Refers to strains that were non-typeable for P; ^5^ Reference category: Pre-vaccine; Bold: The most prevalent genotypes per period.

**Table 3:** G/P type combinations prevalent at Mavalane General Hospital pre- and post-vaccine introduction in Mozambique (2012–2019).

^1^ G/P Genotype Combination	^5^ Pre-Vaccine		Post-Vaccine		OR (95% CI)	*p*-Value
	2012–2015		2016–2019			
	n	%	n	%		
G1P[8]	34	**17.0**	9	**20.9**	1.29 (0.50–3.07)	0.540
G9P[8]	57	**28.5**	1	2.3	0.06 (0.002–0.40)	< 0.001
G12P[6]	26	**13.0**	0	0.0	-	-
G2P[4]	20	10.0	1	2.3	0.21 (0.01–1.42)	0.100
G12P[8]	6	3.0	0	0.0	-	-
G3P[4]	0	0.0	8	**18.6**	-	-
G3P[8]	0	0.0	11	**25.6**	-	-
G8P[4]	5	2.5	1	2.3	0.93 (0.02–8.61)	0.950
G9P[4]	0	0.0	2	4.7	-	-
G9P[6]	0	0.0	2	4.7	-	-
^2^ Other genotypes	5	2.5	3	7.0	2.93 (0.43–15.65)	0.140
^3^ Mixed types	13	6.5	1	2.3	0.34 (0.01–2.41)	0.290
^4^ Partial G/P types	20	10.0	2	4.7	0.44 (0.05–1.93)	0.270
Untypeables	14	7.0	2	4.7	0.64 (0.07–3.00)	0.570
Total	200	100.0	43	100.0	-	-

^1^ It is not possible to calculate the Odds-ratio (OR) for cells with a value of 0; ^2^ Other genotypes: 2012–2015: G12P[4] (0.5%), G2P[6] (1.0%), G2P[8] (0.5%), G8P[8] (0.5%); 2016–2019: G1P[4] (2.3%), G3P[6] (2.3%), G12P[4] (2.3%); ^3^ Mixed types: 2012–2015: G12G8P[4] (1.0%), G12G8P[6] (0.5%), G12G8P[6]P[4] (0.5%), G12G9P[6] (0.5%), G12G9P[8]P[6] (1.0%), G12P[8]P[6] (1.0%), G9G2P[4] (0.5%), G9G2P[6] (0.5%), G9G2P[8] (0.5%), G9P[8]P[4] (0.5%); 2016–2019: G12G3P[4] (2.3%); ^4^ Partial G/P types: 2012–2015: G12P[x] (1.0%), G2P[x] (1.0%),G9P[x] (1.5%), GxP[4] (1.0%), GxP[6] (0.5%), GxP[6]P[4] (0.5%), GxP[8] (4.0%), GxP[8]P[6] (0.5%); 2016–2019: GxP[4] (2.3%),GxP[8] (2.3%); ^5^ Reference category: Pre-vaccine; Bold: The most prevalent genotypes per period.

**Table 4:** G/P type combinations prevalent at five sentinel sites in Mozambique during surveillance pre- and post-vaccine introduction (2015–2019).

^1^ G/P Genotype Combination	^5^ Pre-Vaccine		Post-Vaccine		OR (95% CI)	*p*-Value
	2015		2016–2019			
	n	%	n	%		
G1P[8]	66	**31.0**	40	**20.6**	0.58 (0.36–0.93)	0.020
G3P[4]	0	0.0	28	**14.4**	-	-
G3P[6]	0	0.0	3	1.5	-	-
G3P[8]	0	0.0	19	9.8	-	-
G8P[4]	0	0.0	3	1.5	-	-
G9P[4]	0	0.0	24	**12.4**	-	-
G9P[6]	0	0.0	17	8.8	-	-
G2P[4]	1	0.5	3	1.5	-	-
G2P[6]	9	4.2	8	4.1	0.97 (0.32–2.91)	0.959
G9P[8]	98	**46.0**	7	3.6	0.04 (0.02–0.10)	<0.001
^2^ Other genotypes	3	1.4	4	2.1	1.47 (0.25–10.18)	0.612
^3^ Mixed types	0	0	12	6.2	-	
^4^ Partial G/P types	23	10.8	18	9.3	0.84 (0.41–1.70)	0.611
Untypeables	13	6.1	8	4.1	0.66 (0.23–1.77)	0.370
Total	213	100.0	194	100.0	-	-

^1^ It is not possible to calculate the Odds-ratio (OR) for cells with a value of 0; ^2^ Other genotypes: 2015: G12P[8] (0.9%), G1P[6] (0.5%); 2016–2019: G12P[4] (0.5%), G12P[8] (0.5%), G1P[4] (1.0%); ^3^ Mixed types: 2016–2019: G12G3P[4] (0.5%),G2G1P[8] (0.5%), G3G1P[8] (2.6%),G9G3P[6] (2.6%); ^4^ Partial G/P types: 2015: G9P[x] (3.3%),GxP[6] (0.5%), GxP[8] (7.0%); 2016–2019: G9P[x] (1.0%),GxP[4] (4.6%), GxP[6] (1.6%), GxP[8] (2.1%); ^5^ Reference category: Pre-vaccine; Bold: The most prevalent genotypes per period.

**Table 5:** Prevalence of G/P type combinations at Mavalane General Hospital in Mozambique by year.

G/P Genotype Combination	2012		2013		2014		2015		2016		2017		2018		2019	
	n	%	n	%	n	%	n	%	n	%	n	%	n	%	n	%
G1P[8]	2	3.0	0	0.0	28	84.8	4	5.3	6	66.7	3	18.8	0	0.0	0	0.0
G9P[8]	1	1.5	0	0.0	0	0.0	56	73.7	0	0.0	1	6.3	0	0.0	0	0.0
G12P[6]	26	38.8	0	0.0	0	0.0	0	0.0	0	0.0	0	0	0	0.0	0	0.0
G2P[4]	5	7.5	16	66.7	0	0.0	0	0.0	0	0.0	0	0	1	9.1	0	0.0
G12P[8]	5	7.5	0	0.0	0	0.0	1	1.3	0	0.0	0	0	0	0.0	0	0.0
G3P[4]	0	0	0	0.0	0	0.0	0	0.0	1	11.1	4	25.0	3	27.3	0	0.0
G3P[8]	0	0	0	0.0	0	0.0	0	0.0	0	0.0	0	0	4	36.4	7	100.0
G8P[4]	5	7.5	0	0.0	0	0.0	0	0.0	0	0.0	0	0	1	9.1	0	0.0
G9P[4]	0	0	0	0.0	0	0.0	0	0.0	0	0.0	2	12.5	0	0.0	0	0.0
G9P[6]	0	0	0	0.0	0	0.0	0	0.0	0	0	0	0	2	18.2	0	0.0
^1^ Other genotypes	3	4.5	0	0.0	0	0.0	2	2.6	1	11.1	2	12.5	0	0.0	0	0.0
^2^ Mixed types	13	19.4	0	0.0	0	0.0	0	0.0	1	11.1	0	0	0	0.0	0	0.0
^3^ Partial G/P types	5	7.5	4	16.7	4	12.1	7	9.2	0	0.0	2	12.5	0	0.0	0	0.0
Untypeables	2	3.0	4	16.7	1	3.0	6	7.9	0	0.0	2	12.5	0	0.0	0	0.0
Total	67	100.0	24	100.0	33	100.0	76	100.0	9	100.0	16	100.0	11	100.0	7	100.0

^1^ Other genotypes: 2012: G12P[4] (1.5%), G2P[8] (1.5%), G8P[8] (1.5%); 2015: G2P[6] (2.6%); 2016: G12P[4] (11.1%); 2017: G1P[4] (6.3%), G3P[6] (6.3%); ^2^ Mixed types: 2012: G12G8P[4] (3.0%), G12G8P[6] (1.5%), G12G8P[6]P[4] (1.5%), G12G9P[6] (1.5%), G12G9P[8]P[6] (3.0%), G12P[8]P[6] (3.0%), G9G2P[4] (1.5%), G9G2P[6] (1.5%),G9G2P[8] (1.5%),G9P[8]P[4] (1.5%); 2016: G12G3P[4] (11.1%); ^3^ Partial G/P types: 2012: G12P[x] (3.0%), GxP[6]P[4] (1.5%), GxP[6]P[4] (1.5%), GxP[8]P[6] (1.5%); 2013: G2P[x] (8.3%), GxP[4] (8.3%); 2014: GxP[6] (3.0%), GxP[8] (9.1%); 2015: G9P[x] (4.0%), GxP[8] (5.3%); 2017: GxP[4] (6.3%), GxP[8] (6.3%); Grey: The most prevalent genotypes per year.

**Table 6:** Prevalence of G/P type combinations at five sentinel sites in Mozambique during surveillance by year.

G/P Genotype Combination	2015		2016		2017		2018		2019	
	n	%	n	%	n	%	n	%	n	%
G1P[8]	66	31.0	17	43.6	20	19.2	0	0.0	3	15.0
G3P[4]	0	0.0	1	2.6	14	13.5	12	38.7	1	5.0
G3P[6]	0	0.0	0	0.0	2	1.9	1	3.2	0	0.0
G3P[8]	0	0.0	0	0.0	0	0	7	22.6	12	60.0
G8P[4]	0	0.0	0	0.0	1	1.0	2	6.5	0	0.0
G9P[4]	0	0.0	3	7.7	20	19.2	1	3.2	0	0.0
G9P[6]	0	0.0	5	12.8	9	8.7	3	9.7	0	0.0
G2P[6]	9	4.2	7	17.9	0	0	1	3.2	0	0.0
G9P[8]	98	46.0	0	0.0	6	5.8	1	3.2	0	0.0
^1^ Other genotypes	4	1.9	3	7.7	3	2.9	1	3.2	0	0.0
^2^ Mixed types	0	0.0	2	5.1	10	9.6	0	0.0	0	0.0
^3^ Partial G/P types	23	10.8	0	0.0	14	13.5	2	6.5	2	10.0
Untypeables	13	6.1	1	2.6	5	4.8	0	0.0	2	10.0
Total	213	100.0	39	100.0	104	100.0	31	100.0	20	100.0

^1^ Other genotypes: 2015: G12P[8] (0.9%), G1P[6] (0.5%), G2P[4] (0.5%); 2016: G12P[4] (2.6%), G2P[4] (5.1%); 2017: G12P[8] (1.0%), G1P[4] (1.9%); 2018: G2P[4] (3.2%); ^2^ Mixed types: 2016: G12G3P[4 (2.6%), G2G1P[8] (2.6%); 2017: G3G1P[8] (3.9%), G9G3P[6] (5.8%); ^3^ Partial G/P types: 2015: G9P[x] (3.3%), GxP[6] (0.5%), GxP[8] (7.0%); 2017: G9P[x] (1.9%), GxP[4] (6.7%), GxP[6] (1.9%), GxP[8] (2.9%); 2018: GxP[4](3.2%), GxP[6] (3.2%); 2019: GxP[4] (5.0%), GxP[8] (5.0%); Grey: The most prevalent genotypes per year.

## Supplementary Materials

The following are available online at https://www.mdpi.com/2076-0817/9/9/671/s1: Table S1: Total number of stool samples collected at sentinel sites in Mozambique during surveillance between May 2014 and December 2019; Table S2: Total number of stool samples collected per sentinel sites in Mozambique during surveillance between May 2014 and December 2019; Table S3: Total number of stool samples collected at Mavalane General Hospital during a cross-sectional study (2012–2013) and the National Surveillance of Diarrhea program (2014–2019); Table S4: Distribution of rotavirus genotypes between geographical regions.
