# Supplementary material for: Molecular Epidemiology of Rotavirus A Strains Pre- and Post-Vaccine (Rotarix®) Introduction in Mozambique, 2012–2019: Emergence of Genotypes G3P[4] and G3P[8]
Source: Pathogens. 2020 Aug 19;9(9):671. doi: 10.3390/pathogens9090671 (PMC7557584; doi:10.3390/pathogens9090671)
Supplement: Supplementary file 1 [file pathogens-09-00671-s001.pdf]

Supplementary Tables

Supplementary Table 1. Total number of stool samples collected at sentinel sites in Mozambique during surveillance between May 2014 and December 2019

[1]Period	Year	Total stool samples	Rotavirus positive		Genotyped samples	
			n	%	n	%
Pre-vaccine	[2]2014	97	39	40.2	33	84.6
	2015	555	225	40.5	213	94.7
Subtotal	-	652	264	40.5	246	93.2
Post-vaccine	2016	359	46	12.8	39	84.8
	2017	363	104	28.7	104	100.0
	2018	223	34	15.2	31	91.2
	2019	139	20	14.4	20	100.0
Subtotal	-	1084	204	18.8	194	95.1
Total	-	1736	468	27.0	440	94.0

[1]Data published previously by de Deus et al., 2018 was from January 2014 to June 2017 and included six sentinel sites. In the current analyses, only data from five sentinel sites were analysed (Maputo Central Hospital was excluded)

[2]Only data for Mavalane General Hospital is available

Supplementary Table 2. Total number of stool samples collected per sentinel sites in Mozambique during surveillance between May 2014 and December 2019

[2]Period	Year	[3]Maputo (**HGM & HJM**)					Nampula (HCN)					Quelimane (HGQ)					[5]Beira (HCB)				
		[1]SS	RVA positive		Genotyped samples		SS	RVA positive		Genotyped samples		SS	RVA positive		Genotyped samples		SS	RVA positive		Genotyped samples	
		-	n	%	n	%	-	n	%	n	%	-	n	%	n	%	-	n	%	n	%
Pre-vaccine	[4]2014	97	39	40.2	33	84.6	0	0	0	0	0	0	0	0	0	0	0	0	0	0	0
	2015	325	142	43.7%	141	99.3	192	67	34.9	59	88.1	20	8	40.0	8	100.0	18	6	33.3	5	83.3
Subtotal	-	422	181	42.9	174	96.1	192	67	34.9	59	88.1	20	8	40.0	8	100.0	18	6	33.3	5	83.3
Post-vaccine	2016	68	9	13.2	9	100.0	198	29	14.6	23	79.3	48	6	12.5	5	83.3	45	2	4.4	2	100.0
	2017	154	37	24.0	37	100.0	120	49	40.8	49	100.0	60	16	26.7	14	87.5	29	4	13.8	4	100.0
	2018	124	20	16.1	18	90.0	55	8	14.5	8	100.0	19	4	21.1	4	100.0	25	2	8.0	1	50.0
	2019	62	7	11.3	7	100.0	50	10	20.0	10	100.0	27	3	11.1	3	100.0	0	0	0	0	0
Subtotal	-	408	73	17.9	71	97.3	423	96	22.7	90	93.8	154	29	18.8	26	89.7	99	8	8.1	7	87.5
Total	-	830	254	30.6	245	96.5	615	163	26.5	149	91.4	174	37	21.3	34	91.9	117	14	12.0	12	80.7

[1]SS-Stool samples; RVA – Group A rotavirus

[2]Data published previously by de Deus et al., 2018 was from January 2014 to June 2017 and included six sentinel sites. In the current analyses, only data from five sentinel sites were analysed (Maputo Central Hospital was excluded)

[3]In Maputo: samples were collected at two sentinel sites (Mavalane General Hospital since May 2014) and Jose Macamo General Hospital (March of 2015), in Nampula (Nampula Central Hospital, since March 2015), in Quelimane (Quelimane General Hospital, since October of 2015) and Beira (Beira Central Hospital, since June of 2015)

[4]Only data for Mavalane General Hospital is available

[5]No, samples were collected in 2019 in Beira

Supplementary Table 3. Total number of stool samples collected at Mavalane General Hospital during a cross-sectional study (2012-2013) and the National Surveillance of Diarrhoea program (2014-2019)

Period	Year	Total stool samples	Rotavirus positive		Genotyped samples	
			n	%	n	%
Pre-vaccine	[1]2012	165	74	44.8	67	90.5
	[2]2013	70	25	35.7	24	96.0
	2014	97	39	40.2	33	84.6
	2015	184	76	41.3	76	100.0
Subtotal	-	516	214	41.5	200	93.5
Post-vaccine	2016	46	9	19.6	9	100.0
	2017	77	12	15.6	11	91.7
	2018	82	16	19.5	16	100.0
	2019	59	7	11.9	7	100.0
Subtotal	-	264	44	16.7	43	97.7
Total	-	780	258	33.1	243	94.2

[1,2]Samples collected in the cross-sectional study during 2012-2013 (de Deus et al., 2018) and genetic characterization of rotavirus strains was published previously (João, et al., 2018)

Supplementary Table 4. Geographical distribution of rotavirus genotypes

Maputo (HGM & HJM)										
	Pre-vaccine		Post-vaccine							
[1]G/P type	2015		2016		2017		2018		2019	
	n	%	n	%	n	%	n	%	n	%
G1P[8]	8	5.7	6	66.7	6	16.2	0	0	0	0.0
G3P[4]	0	0	1	11.1	11	29.7	6	33.3	0	0.0
G3P[8]	0	0	0	0	0	0	6	33.3	7	100.0
G9P[4]	0	0	0	0	2	5.4	0	0	0	0.0
G9P[6]	0	0	0	0	0	0	2	11.1	0	0.0
G2P[6]	3	2.1	0	0	0	0	0	0	0	0.0
G9P[8]	97	68.8	0	0	2	5.4	1	5.6	0	0.0
Other genotypes	3	2.1	1	11.1	5	13.5	3	16.7	0	0.0
Mixed type	0	0	1	11.1	0	0	0	0	0	0.0
Partial G/P types	18	12.7	0	0	6	16.2	0	0	0	0.0
Untypeables	12	8.5	0	0	5	13.5	0	0	0	0.0
Total	141	100.0	9	100.0	37	100.0	18	100.0	7	100.0
**Nampula (HCN)**										
[2]G/P type										
G1P[8]	46	78.0	10	43.5	11	22.4	0	0	3	30.0
G3P[4]	0	0.0	0	0.0	1	2.0	4	50.0	1	10.0
G3P[8]	0	0.0	0	0.0	0	0.0	0	0.0	2	20.0
G9P[4]	0	0.0	3	13.0	16	32.7	1	12.5	0	0.0
G9P[6]	0	0.0	5	21.7	4	8.2	1	12.5	0	0.0
G2P[6]	6	10.2	4	17.4	0	0.0	0	0.0	0	0.0
G9P[8]	1	1.7	0	0.0	1	2.0	0	0.0	0	0.0
Other genotypes	1	1.7	0	0.0	1	2.0	0	0.0	0	0.0
Mixed type	0	0.0	0	0.0	10	20.4	0	0.0	0	0.0
Partial G/P types	4	6.8	0	0.0	5	10.2	2	25.0	2	20.0
Untypeables	1	1.7	1	4.3	0	0.0	0	0.0	2	20.0
Total	59	100.0	23	100.0	49	100	8	100.0	10	100.0
**Quelimane (HGQ)**										
[3]G/P type										
G1P[8]	8	100.0	1	20.0	2	14.3	0	0.0	0	0.0
G3P[4]	0	0.0	0	0.0	1	7.1	1	25.0	0	0.0
G3P[8]	0	0.0	0	0.0	0	0.0	1	25.0	3	100.0
G9P[4]	0	0.0	0	0.0	2	14.3	0	0.0	0	0.0
G9P[6]	0	0.0	0	0.0	5	35.7	0	0.0	0	0.0
G2P[6]	0	0.0	2	40.0	0	0.0	1	25.0	0	0.0
G9P[8]	0	0.0	0	0.0	3	21.4	0	0.0	0	0.0
Other genotypes	0	0.0	1	20.0	0	0.0	1	25.0	0	0.0
Mixed type	0	0.0	1	20.0	0	0.0	0	0.0	0	0.0
Partial G/P types	0	0.0	0	0	1	7.1	0	0.0	0	0.0
Total	8	100.0	5	100.0	14	100.0	4	100.0	3	100.0
**Beira (HCB)**										
[4]G/P type										
G1P[8]	4	80.0	0	0.0	1	25.0	0	0.0	0	0.0
G3P[4]	0	0.0	0	0.0	1	25.0	1	100.0	0	0.0
G2P[4]	0	0.0	1	50.0	0	0.0	0	0.0	0	0.0
G2P[6]	0	0.0	1	50.0	0	0.0	0	0.0	0	0.0
Partial G/P types	1	20.0	0	0.0	2	50.0	0	0.0	0	0.0
Total	5	100.0	2	0.0	4	100.0	1	100.0	0	0.0

[1] Maputo: 2015 – Other genotypes: G12P[8] (1.4%), G2P[4] (0.7%), Partial G/P types: G9P[x] (5.0%), GxP[6] (0.7%), GxP[8] (7.1%); 2016 – Other genotypes : G12P[4] (11.1%), Mixed type : G12G3P[4]; 2017- Other genotypes : G1P[4] (5.4%), G3P[6] (5.4%), G8P[4] (2.7%); 2018 - Other genotypes: G2P[4] (5.6%), G8P[4] (11.1%)

[2] Nampula: 2015 – Other genotypes: G1P[6] (1.7%); 2017 – Other genotypes: G12P[8] (2.0%); Mixed type: G3G1P[8] (8.2%), G3G9P6 (12.2%); Partial G/P types: G9P[x] (2.0%), GxP[6] (2.0%), GxP[4] (6.1%); 2018 – Other genoPartial G/P types: GxP[4] (12.5%), GxP[6] (12.5%); 2019 - Partial G/P types: GxP[4] (10.0%), GxP[8] (10.0%)

[3]Quelimane: 2016 – Other genotypes: G2P[4] (20.0%); Mixed type: G2G1P[8] (20.0%); 2017 – Partial G/P type: GxP[6] (7.1%); 2018 - Other genotypes: G3P[6] (25.0%)

[4]Beira: 2015 - Partial G/P types: GxP[8] 1 (20.0%); 2017 - Partial G/P types: G9P[x] (25.0%), GxP[8] (25.0%)
